# Transforming tuberculosis diagnosis with clinical metagenomics: progress and roadblocks

**DOI:** 10.1128/jcm.00537-25

**Published:** 2025-09-19

**Authors:** Yuqing Chen, Hui Tang, Jieyuan Zheng, Qing Yang, Dongsheng Han

**Affiliations:** 1 Department of Laboratory Medicine, the First Affiliated Hospital, Zhejiang University School of Medicine 26441, Hangzhou, People's Republic of China; 2 Zhejiang Key Laboratory of Clinical In Vitro Diagnostic Techniques, Hangzhou, People's Republic of China; 3 Institute of Laboratory Medicine, Zhejiang University 12377, Hangzhou, People's Republic of China; 4 Department of Pathology, the First Affiliated Hospital, Zhejiang University School of Medicine 26441, Hangzhou, People's Republic of China; Vanderbilt University Medical Center, Nashville, Tennessee, USA

**Keywords:** tuberculosis, metagenomics, sequencing, infectious disease, mNGS

## Abstract

Tuberculosis (TB) remains a leading global infectious killer, yet traditional diagnostic methods are inadequate. Acid-fast staining suffers from low sensitivity, and mycobacterial culture requires prolonged incubation because of the slow growth of *Mycobacterium tuberculosis*. PCR-based molecular assays allow rapid detection, but their capacity for resistance profiling is limited to a narrow set of mutations. Metagenomic next-generation sequencing (mNGS) has emerged as a promising culture-independent tool for TB detection, enabling broad-spectrum pathogen identification and offering added value in complex scenarios including extra-pulmonary disease, mixed infections, and infections in immunocompromised or pediatric populations. Clinical studies indicate that mNGS achieves moderate to high sensitivity and excellent specificity in the diagnosis of tuberculosis. However, its diagnostic performance is often constrained by low mycobacterial read counts, interference from abundant host nucleic acids, and the inability to distinguish active from latent infection. In addition, the accuracy of drug resistance prediction using mNGS remains limited, and the World Health Organization currently endorses targeted NGS as the preferred sequencing-based approach for resistance profiling. Despite these challenges, mNGS has facilitated novel diagnostic strategies that combine pathogen detection with host-response data, thereby broadening its potential clinical utility. Nevertheless, practical barriers such as high cost, complex laboratory workflows, and difficulties in data interpretation continue to restrict widespread adoption in routine practice. Future efforts should prioritize technical optimization, standardized protocols, and integration with conventional diagnostics to establish cost-effective and clinically meaningful roles for mNGS in TB diagnosis and management.

## INTRODUCTION

Tuberculosis (TB) continues to represent the foremost cause of mortality among infectious diseases on a global scale. According to data released by the World Health Organization (WHO), an estimated 10.8 million new cases of TB were reported worldwide in 2023, culminating in approximately 1.3 million deaths (equivalent to an average of 3,425 fatalities per day) (1). Although TB is preventable and treatable, existing diagnostic approaches remain encumbered by critical limitations (Table 1). Acid-fast stain (AF stain) fails to detect at least 40.0%–60.0% of pulmonary TB cases (2, 3). The gold-standard culture method is hampered by a protracted turnaround time (TAT). Immunological assays, such as tuberculous infection of T cells spot test (T-SPOT.TB) (Oxford Immunotec, Abingdon, UK), are useful for screening active and latent infections, but their specificity may be reduced in high-prevalence settings or by cross-reactivity with certain non-tuberculous mycobacteria (notably *M. kansasii, M. marinum,* and *M. szulgai*), while their sensitivity can be limited in immunocompromised hosts, early infection, or when samples are poorly handled (4, 5). Rapid molecular assays, including Xpert MTB/RIF (Cepheid, Sunnyvale, CA, USA), TrueNat assays (Molbio Diagnostics, Goa, India), line probe assays (LPA), and loop-mediated isothermal amplification (LAMP) assays, can deliver preliminary results within hours but have limited capacity for comprehensive drug resistance profiling and reduced diagnostic accuracy for pediatric and extra-pulmonary TB (6). Amidst the increasing prevalence of multidrug-resistant TB with suboptimal treatment coverage (~44.0%) (1), there is an urgent clinical imperative for novel diagnostic tools that combine rapid TAT, high sensitivity for both pulmonary TB and extra-pulmonary TB, and the capacity for full-spectrum drug susceptibility testing (DST).

**TABLE 1:** Summary of TB detection methods: strengths, weaknesses, and applicable scenarios^*a*^

Detection methods	Strengths	Weaknesses	Applicable scenarios
mNGS	Short turnaround time (within 24 h) Unbiased broad-spectrum pathogen detection Increased sensitivity and specificity for pulmonary TB, extra-pulmonary TB and TB in special populations Identify complex mixed infections Interference-free detection performance during short-term (within 3 months) anti-tuberculosis treatment Host transcriptional information to screen early TB biomarker	Expensive cost Intricate operation with multiple factors affecting detection accuracy Potential interference from host nucleic acids Inferior detection speed to rapid molecular techniques Extremely complex result interpretation Inability to differentiate between active/latent infections and pathogenic/colonizing microorganisms Ineffective prediction of antibiotic resistance	Traditional tests are negative but MTBC infection cannot be excluded Suspicion of co-infection with rare or atypical pathogens Clinically suggestive of extra-pulmonary TB, such as disseminated TB, tissue or lymph node TB, and urinary tract TB Critically ill patients, immunocompromised hosts, and patients with complex infections of unknown etiology
tNGS	Simultaneous detection of multiple drug resistance genes Enhanced detection sensitivity and specificity of specific target sequences WHO-approved genotypic drug susceptibility testing for TB	Narrow pathogen detection spectrum Inability to identify novel or rare mutations	Comprehensive assessment of TB drug resistance to rapidly guide individualized treatment
WGS	Provision of whole-genome information and theoretical identification of all drug resistance mutations and strain typing Epidemiological tracing and resistance mechanism studies	Insufficient sensitivity for low-load samples Complex data analysis Longer TAT High cost	Molecular epidemiology and transmission tracing of drug-resistant TB Complex drug-resistant TB cases Research applications
AF stain	Rapid, cheap, and easy to operate High specificity Suitable for large-scale screening	Poor sensitivity, especially for extra-pulmonary TB, pediatric TB, and patients co-infected with HIV Inability to distinguish MTBC from NTM	Preliminary screening in resource-limited settings Rapid identification and follow-up evaluation of infectious patients
Culture	Diagnostic gold standard Ability to distinguish viable bacteria and perform drug susceptibility testing Applicable to pulmonary TB and extra-pulmonary TB	Lengthy TAT (6–8 weeks) Rigorous requirements for laboratory infrastructure	Etiological diagnosis and drug resistance analysis Follow-up management of complex, recurrent, and resistant TB cases
Serological tests (T-SPOT.TB)	Screening for active and latent infections Blood sample testing	Inability to differentiate between active and latent infections Affected specificity by endemic region Inability to directly detect drug resistance	Screening for active and latent infections Auxiliary diagnosis of pulmonary TB and extra-pulmonary TB
Rapid molecular assays (Xpert MTB/RIF)	Rapid (within 2 h) and easy to operate High sensitivity Simultaneous detection of MTBC and rifampicin resistance-associated gene mutations (rpoB)	Inability to perform comprehensive drug resistance profiling Reduced sensitivity for extra-pulmonary TB and low bacterial load samples	Rapid initial screening and drug resistance risk assessment Molecular diagnostics for resource-limited settings

^*a*^ mNGS, metagenomic next-generation sequencing; tNGS, targeted next-generation sequencing; WGS, whole genome sequencing; MTBC, *mycobacterium tuberculosis* complex; NTM, *nontuberculous mycobacteria*; TB, tuberculosis; AF stain, acid-fast stain; T-SPOT. TB, tuberculous infection of T cells spot test; WHO, World Health Organization; TAT, turnaround time.

Next-generation sequencing (NGS) offers a promising solution. Current available NGS-based techniques include mycobacterial whole genome sequencing (WGS), targeted NGS (tNGS), and metagenomic NGS (mNGS). WGS can provide exhaustive genomic insights but suffers from limited sensitivity in specimens with low bacterial burden, complex bioinformatic requirements, and prohibitive costs (7). Targeted NGS, endorsed by the WHO in March 2024 for genotypic DST in drug-resistant TB, enhances sensitivity for predefined resistance loci but remains constrained by primer/probe design; mutations in primer or probe binding sites may cause drop-out of certain regions (8). In contrast, mNGS is an unbiased, culture-independent technique that sequences all nucleic acids within a clinical specimen, enabling broad pathogen detection and functional gene analysis (9). Overall, these approaches represent a trade-off: WGS offers complete genomic resolution of cultured isolates; tNGS provides in-depth analysis of specific loci; and mNGS allows broad-spectrum pathogen detection in low-burden or polymicrobial samples.

This study analyzed the published evidence and real-world data to delineate the current capabilities and unresolved challenges of mNGS in TB diagnosis, aiming to furnish clinical microbiologists and physicians with an objective framework for technical evaluation and to guide the rational application of this promising yet nascent technology in clinical practice.

## MNGS: BETWEEN THE IDEAL OF UNBIASED DETECTION AND REAL-WORLD BIASES

mNGS is theoretically a non-targeted, broad-spectrum nucleic acid detection technology capable of identifying all pathogens present in a sample within a single assay, offering the advantage of unbiased detection. Over more than a decade of clinical translation, mNGS has demonstrated capabilities that conventional microbiological methods rarely achieve, particularly in detecting rare or atypical pathogens and diagnosing mixed infections. Clinical studies have shown its broad applicability across diverse specimen types (10). For blood and cerebrospinal fluid (CSF) samples testing negative by conventional methods, mNGS can increase detection rates by approximately 10.0%–40.0% (11) and 5.0%–20.0% (12), respectively, substantially enhancing the diagnosis of complex infections. These advantages have driven its continued adoption and efforts toward standardization in clinical laboratories.

In real-world practice, however, the accuracy and reproducibility of mNGS are influenced by multiple factors, making both false positives and false negatives unavoidable. Common causes of false positives include cross-contamination, background microbial DNA, and species misclassification during bioinformatic analysis, with background microorganisms accounting for 49.0% of false-positive events (13). In a multicenter study, we found that 76.2% of false-negative results were attributable to microbial nucleic acid loss during the “wet lab” phase (14). Such losses may result from operator errors, suboptimal extraction efficiency of commercial kits, host nucleic acid interference, and excessive fragmentation or degradation of microbial nucleic acids. The expertise of personnel in interpreting results is also critical: in our multicenter external quality assessment (EQA) of mNGS (15), we observed that some laboratories detected the relevant pathogen but failed to correctly identify it (false negatives), whereas others misinterpreted contaminants or nonpathogenic microbes as the causative agent (false positives).

These observations underscore the methodological complexity of mNGS and help explain why its implementation under a unified standard across laboratories remains challenging. At present, no mNGS workflow has been approved for use as an *in vitro* diagnostic (IVD) assay; most are offered as laboratory-developed tests (LDTs) within reference or high-capacity laboratories. In practice, strict adherence to validated standard operating procedures (SOPs) is essential to minimize the risk of experimental bias and to realize, as much as possible, the theoretical advantage of unbiased detection that mNGS promises.

## DIAGNOSTIC YIELD OF MNGS-BASED TB DETECTION

### Pulmonary TB

Accumulating clinical evidence underscores the considerable diagnostic value of mNGS in the detection of pulmonary TB. Numerous studies have demonstrated that mNGS can substantially enhance the detection of *Mycobacterium tuberculosis* complex (MTBC) in various clinical specimens, including sputum, bronchoalveolar lavage fluid (BALF), and lung tissue biopsies (Table 2). mNGS demonstrated a sensitivity of 60.0%–83.0%, which was comparable to Xpert MTB/RIF and substantially exceeded that of mycobacterial culture (42.0%–50.0%), while preserving a high specificity of 98.0%–99.0% (16). In a study by Liu et al., mNGS detected MTBC in 33.2% (5/34) of the BALF samples from clinically confirmed TB patients that were negative by both Xpert MTB/RIF and culture (17). When mNGS and traditional diagnostic methods (Xpert MTB/RIF, mycobacterial culture, BALF AF stain, or lung biopsy pathological examination) are used concurrently on the same sample, the diagnostic sensitivity can be further increased to 91.5% (18), consistent with findings from other investigations (17, 19, 20). Furthermore, mNGS can simultaneously detect co-infections involving MTBC alongside fungi, bacteria, and/or viruses in a single assay. It also enables rapid and accurate differentiation between MTBC and nontuberculous mycobacteria (NTM) in AF stain-positive BALF samples, demonstrating its unbiased capacity to identify diverse pathogens (3). Importantly, its diagnostic performance is not compromised by short-term (≤3 months) anti-tuberculosis therapy (17). These technical advantages make it particularly valuable in clinical scenarios involving atypical radiological findings, suspected mixed infections, or complex cases requiring etiological confirmation during anti-TB treatment.

**TABLE 2:** Diagnostic performance of mNGS and other methods for TB: Summary of included studies^*a*^

Study (first author/year)	Sample types	Detection methods	Sensitivity (%, 95% CI, n/N)	Specificity (%, 95% CI, n/N)
Xu P 2022 (3)	BALF (all patients)	mNGS	94.4 (88.9-99.9, 67/71)	100.0 (99.9-100.0, 23/23)
		Xpert MTB/RIF	85.9 (77.6–94.2, 61/71)	100.0 (99.9–100.0, 23/23)
		AF stain	28.2 (17.5–38.9, 20/71)	73.9 (54.5–93.3, 17/23)
		mNGS with Xpert MTB/RIF	97.2 (93.2–100, 69/71)	100.0 (99.9–100.0, 23/23)
	Blood (all patients)	T-SPOT.TB	64.8 (53.4–76.2, 46/71)	91.3 (78.9–100.0, 21/23)
	BALF (immunocompromised patients)	mNGS	93.5 (84.4–100.0, 29/31)	100.0 (99.9–100.0, 5/5)
		Xpert MTB/RIF	80.6 (66.0–95.4, 25/31)	100.0 (99.9–100.0, 5/5)
		AF stain	32.3 (14.8–49.7, 10/31)	40.0 (28.0–100.0, 2/5)
		mNGS with Xpert MTB/RIF	100.0 (99.9–100.0, 31/31)	100.0 (99.9–100.0, 5/5)
	Blood (immunocompromised patients)	T-SPOT.TB	48.4 (29.8–67.0, 15/31)	100.0 (99.9–100.0, 5/5)
Liu X 2021 (17)	BALF	mNGS	59.9 (85/142)	100.0 (111/111)
		Xpert MTB/RIF	69.0 (98/142)	100.0 (111/111)
		Culture	59.9 (85/142)	100.0 (111/111)
		AF stain	24.6 (35/142)	93.7 (104/111)
Xiong W 2025 (18)	BALF and lung biopsy tissue	mNGS	74.2 (63.9–82.5, 69/93)	97.0 (91.9–99.0, 127/131)
		AF stain	41.9 (31.9–52.6, 39/93)	93.9 (87.9–97.1, 123/131)
	BALF and lung biopsy tissue	mNGS	74.5 (64.3–82.7, 70/94)	96.97 (92.0–99.0, 128/132)
		mNGS with Xpert MTB/RIF, culture, AF stain, and pathology	91.5 (83.4–96.0, 86/94)	90.9 (84.3–95.0, 120/132)
	BALF	mNGS	74.4 (64.0–82.8, 70/94)	96.9 (91.7–99.0, 124/128)
		Culture	47.8 (37.2–58.5, 43/90)	100.0 (96.4–100.0, 128/128)
Xiao H 2025 (19)	BALF	mNGS	94.6 (106/112)	98.9 (186/188)
		Xpert MTB/RIF	44.6 (50/112)	87.2 (164/188)
		Culture	44.6 (50/112)	94.7 (178/188)
		AF stain	26.8 (30/112)	94.7 (178/188)
Ou Y 2025 (20)	BALF	Nanopore sequencing	98.7 (92.0–99.9, 76/77)	96.2 (78.4–99.8, 25/26)
		TB-DNA	44.2 (33.0–55.9, 34/77)	96.2 (78.4–99.8, 25/26)
		Culture	18.1 (10.6–29.0, 14/77)	100.0 (84.0–100.0, 26/26)
Jin W 2020 (21)	Sputum	mNGS	52.3 (31.1–72.6, 12/23)	98.5 (96.0–99.5, 265/269)
		Culture	60.9 (38.8–79.5, 14/23)	100.0 (98.2–100.0, 269/269)
	BALF	mNGS	55.0 (32.0–76.2, 11/21)	98.2 (93.0–99.7, 108/110)
		Culture	28.6 (12.2–52.3, 6/21)	100.0 (95.8–100.0, 110/110)
	Lung biopsy tissue	mNGS	88.9 (50.7–99.4, 8/9)	97.8 (86.7–99.9, 44/45)
		Culture	22.2 (3.0–59.8, 2/9)	100.0 (90.2–100.0, 45/45)
	Serous fluid	mNGS	50.0 (32.8–67.2, 17/34)	97.3 (84.2–99.9, 36/37)
		Culture	23.5 (11.4–41.6, 8/34)	100.0 (88.3–100.0, 37/37)
	Pus	mNGS	50.0 (20.1–79.9, 5/10)	98.0 (88.2–99.9, 50/51)
		Culture	50.0 (20.1–79.9, 5/10)	100.0 (91.3–100.0, 51/51)
	Tissue	mNGS	40.9 (21.4–63.3, 9/22)	100.0 (82.2–100.0, 23/23)
		Culture	40.9 (21.4–63.3, 9/22)	100.0 (82.2–100.0, 23/23)
	Blood	mNGS	0.0 (0.0–69.0, 0/3)	100.0 (95.3–100.0, 99/99)
		Culture	0.0 (0.0–69.0, 0/3)	100.0 (95.3–100.0, 99/99)
	CSF, swab, secretion, bone marrow, bile, urine, paraffin section, and saliva	mNGS	0.0 (0.0–69.0, 0/3)	95.1 (85.4–98.7, 58/61)
		Culture	0.0 (0.0–69.0, 0/3)	100.0 (92.6–100.0, 61/61)
Fu M 2022 (22)	BALF, lung biopsy tissue	mNGS	78.3 (65.9–90.7, 36/46)	100.0 (36/36)
		Xpert MTB/RIF	76.1 (63.3–88.9, 35/46)	100.0 (35/35)
Wang S 2019 (23)	CSF	mNGS	66.7 (8/12)	100.0 (6/6)
		Xpert MTB/RIF	25.0 (3/12)	100.0 (6/6)
		Culture	8.3 (1/12)	100.0 (6/6)
		AF stain	33.3 (4/12)	100.0 (6/6)
Sun W 2021 (24)	CSF, pleural effusion, ascites, pericardial effusion, articular cavity effusion, pus, lymph node biopsy	mNGS	56.1 (48.53–63.43, 101/180)	100.0 (85.0–100.0, 28/28)
		Xpert MTB/RIF	36.1 (29.2–43.6, 65/180)	96.4 (79.8–99.8, 27/28)
		Culture	13.9 (9.4–20.0, 25/180)	100.0 (83.4–100.0, 27/27)
		Xpert MTB/RIF and culture	37.8 (30.8–45.3, 68/180)	96.4 (79.8–99.8, 27/28)
Ma J 2024 (25)	Blood	mNGS	67.8 (19/28)	100.0 (20/20)
		T-SPOT.TB	82.1 (23/28)	80.0 (16/20)
Yuan J 2024 (26)	BALF	mNGS	58.6 (40.7–76.5, 17/29)	96.8 (90.6–100, 30/31)
		Culture	42.6 (29.4–55.8, 23/54)	100.0 (100.0–100.0, 62/62)
		Xpert MTB/RIF, AF stain and T-SPOT.TB	38.9 (25.9–51.9, 21/54)	100.0 (100.0–100.0, 62/62)
		Nanopore sequencing	68.0 (55.1–80.9, 34/50)	100.0 (100.0–100.0, 51/51)
Zhao H 2024 (27)	BALF	mNGS	46.5 (32.5–61.1, 20/43)	100.0 (84.5–100.0, 21/21)
		TB-PCR	55.0 (39.8–69.3, 22/40)	100.0 (72.3–100.0, 10/10)
		Xpert MTB/RIF	55.0 (34.2–74.2, 11/20)	88.9 (56.5–99.3, 8/9)
		mNGS with TB-PCR	62.8 (47.9–75.6, 27/43)	100.0 (84.5–100.0, 21/21)
Yuan J 2025 (28)	BALF	Host DNA depletion-assisted mNGS	72.0 (60.8–81.0, 59/82)	87.5 (60.8–81.0, 14/16)
		Host DNA depletion-assisted nanopore sequencing	58.5 (47.1–69.1, 48/82)	81.3 (47.1–69.1, 13/16)
		mNGS	51.2 (40.0–62.3, 42/82)	93.8 (15/16)
		Xpert MTB/RIF	49.4 (38.0–60.8, 39/79)	100.0 (12/12)
		Culture	32.5 (22.5–44.2, 25/77)	100.0 (13/13)

^*a*^ mNGS, metagenomic next-generation sequencing; AF stain, acid-fast stain; T-SPOT. TB, tuberculous infection of T cells spot test; TB, tuberculosis; BALF, bronchoalveolar lavage fluid; CSF, cerebrospinal fluid; CI, confidence interval.

However, two major clinical concerns remain regarding mNGS application in pulmonary TB diagnosis: sensitivity limitations and specificity/interpretation challenges.

#### Sensitivity limitations

The intrinsic biological characteristics of MTBC, such as its intracellular localization and thick cell wall, in conjunction with the limited sequencing depth of traditional mNGS platforms (typically ≤20 million short reads per sample) and their susceptibility to interference from host nucleic acids, may collectively lead to suboptimal detection sensitivity. In the course of clinical practice at our laboratory from February 2012 to February 2025, we observed that among 1,056 mNGS-confirmed pulmonary TB cases, a substantial proportion of samples exhibited low MTBC read counts, with 37.0% (391/1,056) having reads per million (RPM) ≤10, 23.0% (242/1,056) with RPM ≤3, and 12.0% (127/1,056) with only a single read (RPM = 1). Similarly, Jin et al. reported that approximately 50.0% of TB-positive results presented with only 1–5 reads (21). Such low read counts may reflect a combination of host nucleic acid interference, low bacterial burden, and technical factors such as nucleic acid extraction efficiency, or alternatively, arise from background contamination introduced during sample processing or sequencing reagents. Therefore, positive results with very low read counts, particularly RPM <3 or a single read, must be interpreted in the context of clinical manifestations, additional laboratory findings, and relevant epidemiological information to minimize the risk of misdiagnosis or missed diagnosis. Currently, no universally accepted threshold for sequencing read counts exists; accordingly, to ensure optimal sensitivity, many clinical laboratories in China consider a single species-specific read sufficient for a positive report, provided that internal quality control standards are met (29).

#### Specificity and interpretation challenges

Studies have shown that mNGS demonstrates high diagnostic specificity (≥90.0%) in TB patients (Table 2). This advantage is primarily attributable to the fact that MTBC is a well-defined human pathogen, neither part of the normal commensal microbiota nor a common laboratory background contaminant. Therefore, the detection of MTBC sequences usually indicates the genuine presence of the organism or its nucleic acids in the patient (21). However, when applied to pathogens other than MTBC, the diagnostic specificity of mNGS is relatively limited. For example, the Karius test (Karius, Redwood City, CA, USA), which utilizes plasma cell-free DNA for NGS-based pathogen detection, has a reported diagnostic specificity of only 63.5% (30). Nearly half of the positive results were polymicrobial, yet these did not always correspond to clinically significant infections (31). Nucleic acids detected in blood or plasma may originate from translocation of commensal organisms across impaired mucosal barriers or from exogenous contamination introduced during laboratory procedures, such as the amplification of background microbial DNA present in reagents, which can result in false-positive findings. The situation is even more complex in respiratory specimens. The upper respiratory tract harbors a dense community of commensals that inevitably contribute to background signals, and additional microbial DNA may be introduced during sample collection and processing. Consequently, mNGS reports from respiratory specimens often reveal highly complex microbial profiles, making it difficult for clinicians to pinpoint the causative pathogen responsible for secondary or mixed infections in TB patients. This is particularly problematic when interpreting the clinical significance of opportunistic organisms, which must be carefully interpreted within the context of each patient’s specific condition. It should also be emphasized that, similar to commonly used molecular assays such as Xpert MTB/RIF, mNGS detects microbial nucleic acid fragments rather than viable organisms. Therefore, it cannot distinguish between live and dead bacilli, nor can it differentiate latent from active TB. Given the low bacterial load and dormant state associated with latent TB infection, mNGS based solely on DNA detection is currently not suitable for definitive diagnosis of latent TB. Assessment of disease activity still requires integration of imaging findings, complementary diagnostic tests (e.g., culture, T-SPOT.TB), and clinical history. Looking ahead, whether specific biomarkers associated with latent infection can be extracted from the rich mNGS data sets represents an important area of exploration and holds promise for expanding the clinical utility of mNGS (32).

Choosing the appropriate respiratory specimen is critical for the accurate diagnosis of pulmonary TB. Common clinical specimen types such as BALF, sputum, and lung biopsy tissue differ significantly in pathogen load and detection sensitivity, which, in turn, affects the diagnostic performance of mNGS. Although sputum samples are easy to obtain, their high content of PCR inhibitors and complex commensal microbiota result in a markedly lower mNGS sensitivity compared to BALF samples (16, 33). By contrast, BALF can reflect pathogens in the lower respiratory tract more accurately and are generally considered a more suitable specimen type for diagnosing pulmonary infections. Lung biopsy tissues, which are obtained directly from the lesion site, typically contain a higher pathogen burden and fewer background microbes, leading to significantly improved mNGS sensitivity for detecting MTBC compared with BALF (21, 22). This characteristic makes lung biopsy specimens particularly valuable in patients with peripheral pulmonary lesions (PPLs) or those for whom bronchoalveolar lavage is contraindicated. In clinical practice, specimen selection should be guided by the patient’s condition and sample availability, with preference given to the type offering the greatest diagnostic value. When necessary, testing multiple specimen types in combination may further improve diagnostic accuracy.

### Extra-pulmonary TB

mNGS is also effective for the diagnosis of extra-pulmonary TB, as studies have shown its unique value as a targeted diagnostic adjunct across diverse extra-pulmonary specimens with high pre-test probability of TB. Ai et al. reported a case in which mNGS of liver biopsy tissue provided a definitive diagnosis of hepatic TB despite complex imaging findings and negative traditional pathology (H&E and Ziehl-Neelsen staining) (34). In tuberculous meningitis, Wang et al. found that mNGS performed on CSF samples achieved significantly higher sensitivity (66.7%) than AF stain (33.3%), PCR (25.0%), and culture (8.3%), while also enabling the simultaneous detection of mixed infectious pathogens, such as *Cryptococcus neoformans* (23). Sun et al. evaluated smear-negative extra-pulmonary specimens (CSF, serous effusions, pus, lymph node tissue, and urine) and reported overall mNGS sensitivity of 56.1%, substantially higher than Xpert MTB/RIF (36.1%) and BACTEC Mycobacteria Growth Indicator Tube (MGIT) 960 (Becton Dickinson, Sparks, MD, USA) (13.9%), with tuberculous meningitis detection up to 84.4% (24). Notably, this study found that anti-tuberculosis therapy had a relatively minor impact on mNGS, with the positive rate decreasing from 62.8% to 52.9%, whereas the detection rates of Xpert MTB/RIF (41.9% to 23.5%) and MGIT 960 (19.5% to 0.0%) declined substantially during treatment. This finding is consistent with Liu et al.’s study showing that mNGS detection in pulmonary TB is less affected by anti-tuberculosis drugs (17), indicating that mNGS maintains robust diagnostic performance even during treatment and thus offers a broader window for clinical sampling. Importantly, mNGS can also uncover MTBC in specimens from patients in whom TB is not initially on the differential. For example, in a seven-year longitudinal monitoring study from the University of California, San Francisco (UCSF), Wilson et al. detected low-abundance MTBC reads in CSF from patients without prior suspicion of TB, confirming mNGS’s hypothesis-free capability to identify occult tuberculosis infections (35). In sum, these advantages have established mNGS as both a powerful adjunct in high-suspicion cases and an agnostic tool for revealing extra-pulmonary TB and monitoring patients receiving anti-tuberculosis therapy.

However, the application of mNGS in diagnosing extra-pulmonary TB still has certain limitations, as its sensitivity is affected by multiple factors. The performance validation of Karius test demonstrated that sequencing depth and the concentration of human DNA in samples are key factors influencing MTBC detection sensitivity-the higher the human cell content and the lower the sequencing depth, the poorer the sensitivity (30). In our study of 508 patients with tissue samples tested, mNGS successfully diagnosed 60 tuberculosis patients (which typically have high human cell content), there were still four missed cases (two identified by tissue PCR and two by GeneXpert MTB/RIF tests) (36). Similarly, a 7-year longitudinal monitoring study from the UCSF mNGS laboratory found that due to the paucibacillary nature of CSF samples in tuberculous meningitis, 92.3% (12/13) of MTBC-positive cases had sequence reads below the preset positive threshold, forcing the lab to lower the reporting threshold to improve sensitivity (35). Furthermore, compared to tuberculous meningitis, tuberculous lymphadenitis, and osteoarticular TB, mNGS demonstrated inferior diagnostic performance in serous cavity effusions, including pleural, peritoneal, and pericardial fluids (24). Our clinical observations also revealed that even when pleural fluid adenosine deaminase levels were significantly elevated or sputum was positive for AF stain in patients with tuberculous pleuritis, the positivity rate of mNGS in pleural fluid remained unsatisfactory. This may be due to the presence of PCR inhibitors in serous effusions that interfere with molecular detection accuracy (37). Therefore, unless there is purulent involvement, fresh or frozen biopsy tissue from the lesion site is recommended over serous effusions as the preferred sample for testing.

### TB in special populations

TB diagnosis in immunocompromised populations, such as individuals with HIV infection, presents significant clinical challenges. These patients are more susceptible to developing extra-pulmonary TB or disseminated TB, often characterized by low bacterial burden, complex mixed infections, and atypical clinical manifestations, all of which complicate diagnosis and reduce the effectiveness of traditional diagnostic methods (38). A study demonstrated that plasma mNGS achieved a detection rate of 67.9% among 28 disseminated TB patients, with MTBC being more readily identified in those with elevated procalcitonin (PCT) levels, HIV infection, and decreased CD4+ T cell counts (25). Likewise, Guo et al. revealed that mNGS enables earlier detection of active TB following allogeneic hematopoietic stem cell transplantation compared to traditional methods, thereby facilitating prompt initiation of anti-tuberculosis therapy (39). Furthermore, immunocompromised patients, including those with HIV, frequently present with multiple opportunistic infections. mNGS can simultaneously identify MTBC and a broad spectrum of co-infecting pathogens, such as *Pneumocystis jirovecii*, *Talaromyces marneffei*, *Aspergillus fumigatus*, NTM, and *human herpesviruses*, thus providing essential information for individualized treatment (26).

Pediatric TB remains a major public health challenge as well. According to WHO data, approximately 1.3 million children and adolescents (0–14 years) developed TB in 2023, accounting for 12.0% of all cases (17). The clinical manifestations of pediatric TB are often age-dependent, with younger high-risk children more frequently presenting with paucibacillary intrathoracic or disseminated disease, whose positive rate of culture was only 25.0%– 40.0%. Diagnostic difficulties are further compounded by challenges in specimen collection and the typically low bacterial burden in pediatric cases (40). The clinical features of pediatric pulmonary TB often overlap with those of other bacterial or viral pneumonia, increasing the risk of misdiagnosis and delayed or inappropriate treatment (41). Current research on mNGS for pediatric TB diagnosis is limited. A study found that the sensitivity of mNGS in BALF samples from children with pulmonary TB was 46.5% (20/43), slightly lower than that of TB-PCR and Xpert MTB/RIF (both 55.0%, 22/40), but significantly higher than traditional culture and AF stain (27). Importantly, combining mNGS with TB-PCR increased diagnostic sensitivity to 62.8% (27), highlighting the benefit of multi-modality testing strategies for improving early diagnosis in pediatric TB.

## TB DRUG RESISTANCE PREDICTION: LOFTY IDEALS MEET HARSH REALITY

Drug resistance in MTB has become a pressing global public health concern. In 2023, there were an estimated 400,000 cases of multidrug-resistant or rifampicin-resistant TB worldwide (1), and the 2024 WHO Bacterial Priority Pathogens List designates MTB as a pathogen of critical priority (42). Rapid detection of drug resistance genes allows for earlier and individualized treatment in contrast to traditional DST, which is time-consuming. The Xpert MTB/RIF assay can simultaneously identify MTB and rifampicin resistance-associated mutations (rpoB gene) within 2 h, but it does not cover other key resistance mutations required for full drug susceptibility profiling. In theory, mNGS can directly detect resistance genes from clinical samples and assist in predicting drug resistance; however, its accuracy for comprehensive drug resistance prediction remains unproven. The main reasons may be as follows. First, MTBC is a typical thick-walled bacterium with low nucleic acid extraction efficiency, resulting in a limited yield of target sequences during sequencing and insufficient genome coverage for drug-resistance mutation analysis. Second, there are inherent limitations in sequencing technologies. Short-read platforms such as Illumina typically generate reads no longer than 300 bp, which is often insufficient to span most mobile genetic elements, while long-read platforms such as nanopore sequencing are associated with relatively high base error rates. Third, most resistance mechanisms in MTBC are mediated by single nucleotide variants, which require much higher sequencing depth (10×−100×) than species identification in order to accurately detect mutation signals (43). Fourth, the incompleteness and infrequent updating of resistance mutation databases, as well as the lack of standardized bioinformatics analysis pipelines, further restrict the application of mNGS in predicting drug resistance in TB (44). These challenges highlight the need for further optimization of sample preprocessing, increased sequencing depth, and the development of targeted enrichment techniques to enhance the reliability of mNGS-based drug resistance prediction. One study introduced a novel host DNA depletion-assisted mNGS technique, which reportedly boosts MTBC genome coverage by roughly 16-fold and markedly improves detection of drug-resistance mutations; however, its clinical utility remains to be established (28).

Notably, tNGS based on PCR amplification or probe enrichment techniques has enabled the simultaneous assessment of resistance to multiple anti-tuberculosis drugs within a single test platform. Meta-analyses have shown that the sensitivity of tNGS for resistance testing to individual drugs ranged from 76.5% (capreomycin, 52.5%–92.3%) to 99.1% (rifampicin, 98.3%–99.7%), while specificity ranged from 93.1% (ethambutol, 88.0%–96.3%) to 99.4% (amikacin, 98.3%–99.8%) (45). For rifampicin resistance, the sensitivity of tNGS (99.1%) was higher than that reported for Xpert MTB/RIF Ultra (95.0%), with comparable specificity (97.6% vs 98.0%). For isoniazid, fluoroquinolones, and injectable drugs, the sensitivity and specificity of tNGS are also analogous to those reported for Xpert MTB/XDR. The WHO officially endorsed tNGS for genotypic DST in March 2024 and identified it as a priority for the development of new TB diagnostic tool (8). Given that mNGS currently lacks sufficient capability for comprehensive resistance gene detection in TB, tNGS can be regarded as the most efficient sequencing approach at this stage for rapid and systematic evaluation of tuberculosis drug resistance.

## BREAKING BOUNDARIES: MNGS EMPOWERS A NEW MICROBE-HOST INTEGRATED DIAGNOSTIC PARADIGM FOR TB

mNGS is fundamentally an omics-based technology, generating vast amounts of sequencing data in which microbial reads typically account for less than 5.0%, even below 1.0%, while the overwhelming majority originate from the human genome. Extracting disease-relevant host biomarkers from the abundant human-derived sequences has emerged as a key area of research. In recent years, integrated diagnostic models combining microbial and host response profiles derived from mNGS data have begun to emerge in the field of infectious disease diagnosis (46, 47). In the field of TB diagnosis, Ramachandran et al. developed a machine learning model for tuberculous meningitis by integrating mNGS-based pathogen profiles and host differential gene expression data from CSF of 368 HIV-positive patients with subacute meningitis. The model achieved a sensitivity of 88.9% and a specificity of 86.7%, effectively distinguishing TB meningitis from other clinically similar conditions (48). This innovative study overcame the limitations of relying solely on pathogen detection or host biomarkers, offering a novel strategy for the precise diagnosis of TB. Xu et al. utilized copy number variation (CNV) features from host-derived sequences in pleural effusion mNGS data to predict malignancies and distinguish them from tuberculous pleurisy (49). Our team has also developed a novel AI-assisted diagnostic strategy based on mNGS data from BALF samples. This approach integrates multiple dimensions of information, including microbial DNA and RNA composition, bacteriophage abundance, host gene and transposable element expression levels, immune cell composition, and tumor fraction derived from CNV, to differentiate pulmonary infections, including pulmonary TB, from lung cancer (50). The diagnostic strategy achieved an area under the receiver operating characteristic curve (AUC) of 0.9 (95% CI  =  0.9–1.0) in the training cohort and 0.8 (95% CI  =  0.8–0.9) in the validation cohort. These research efforts have significantly expanded the application scope of mNGS. Although the accuracy and generalizability of such tools remain limited by factors such as study population, analytical methods, and data quality, the integration of multidimensional biological information from both pathogen and host responses holds promise for providing a more comprehensive representation of disease states. This approach offers strong potential to support precision diagnostics for tuberculosis and other complex infectious diseases, and represents a direction worthy of continued investigation.

## PRACTICAL CHALLENGES: CLINICAL TRANSLATION OF MNGS

Despite the broad detection range and high sensitivity of mNGS, which have significantly improved the detection rate of MTBC, this emerging technology still faces several practical challenges in clinical application.

**Fig 1:**
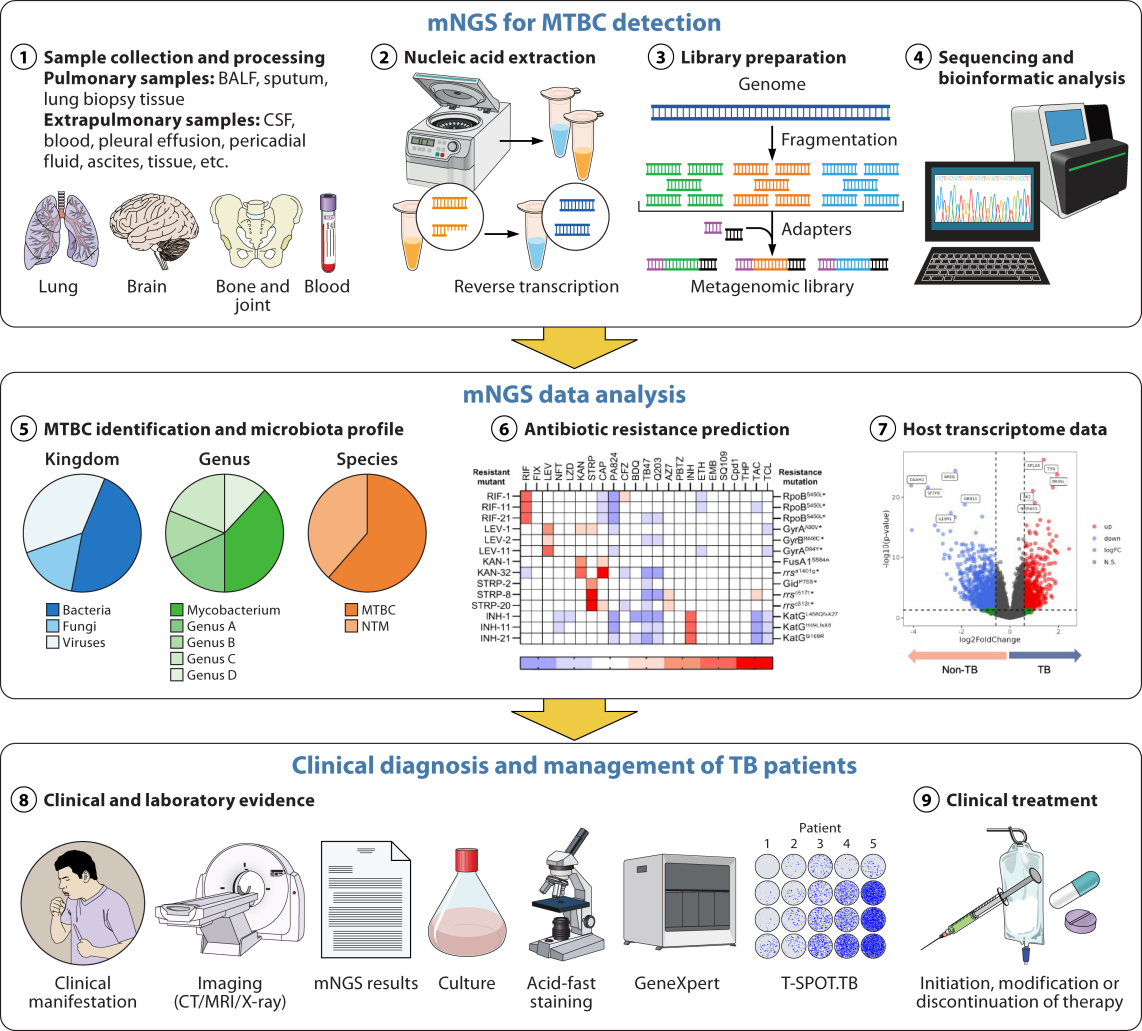
Schematic diagram of mNGS worflow for MTBC detection, mNGS data analysis, and clinical diagnosis/management of TB patients. Abbreviations: mNGS, metagenomic next-generation sequencing; MTBC, *mycobacterium tuberculosis* complex; NTM, nontuberculous mycobacteria; TB, tuberculosis; BALF, bronchoalveolar lavage fluid; CSF, cerebrospinal fluid.

From a technical perspective, traditional mNGS is an intricate omics-based method. The wet-lab workflow involves multiple delicate steps, including sample preprocessing, nucleic acid extraction, library preparation, and high-throughput sequencing (Fig. 1). These procedures encompass centrifugation, extraction, filtration, and purification, which mostly involve low-input operations requiring advanced technical skills and strict adherence to standardized protocols. However, the time-intensive and complex nature of these procedures makes it challenging for laboratory personnel to sustain optimal performance throughout, increasing the risk of human error and compromising result accuracy.

On the dry-lab side, bioinformatics infrastructure is equally critical and challenging. High-performance computing hardware (servers or clusters), validated and automated data processing pipelines for quality control, host read depletion, pathogen identification, and resistance mutation analysis, as well as curated, regularly updated reference databases form the foundation. Skilled bioinformaticians are also essential to manage data analysis, interpret clinical results, and maintain data security and compliance. In laboratories with limited staff or underdeveloped bioinformatics infrastructure, sustaining consistent mNGS operations can be a considerable challenge.

While automated platforms can help minimize manual errors, reduce batch-to-batch variability, and streamline workflows, two major technical hurdles remain. First, the system must offer high compatibility and flexibility to accommodate customized protocols for diverse sample types, ensuring optimal nucleic acid extraction and library preparation across a broad range of pathogens. Second, because mNGS is highly sensitive to exogenous nucleic acid contamination, platform design must address how to effectively prevent cross-contamination from robotic arms and aerosol exposure—an issue best mitigated through the use of fully enclosed automated systems. Notably, such advanced laboratory infrastructure and bioinformatics capacity are often lacking in the regions where MTBC is most highly endemic, further complicating large-scale implementation.

Financial constraints further limit mNGS’s widespread clinical adoption. Significant capital investment is required for sequencing instruments and computational infrastructure, while consumables (including reagents, extraction kits, library preparation materials, and sequencing chips) add ongoing costs. Skilled laboratory technicians and bioinformaticians represent further labor expenses, alongside expenses for ongoing training, quality assurance, and data storage infrastructure. Compared with conventional diagnostics like AF stain, culture, and molecular assays, mNGS remains relatively expensive on a per-sample basis. However, economies of scale, automation, and multiplexing strategies can significantly reduce costs in high-throughput centers. Importantly, economic analyses should consider clinical benefits such as earlier and more accurate diagnosis that may offset increased laboratory expenses. In low-resource settings, innovative solutions like centralized testing hubs, subsidized platforms, and collaborative networks are vital to improve cost-effectiveness and expand access.

Moreover, robust validation is crucial to ensure the accuracy, reliability, and reproducibility of mNGS assays. Analytical validation should establish the limits of detection, sensitivity, specificity, precision, and reproducibility using standardized reference materials and simulated samples representing a range of pathogen loads. Clinical validation requires performance assessment across varied specimen types and populations. Quality control throughout pre-analytical, analytical, and post-analytical phases is essential to minimize errors and contamination. Harmonizing protocols with international standards, such as WHO recommendations and CLSI guidelines, enhances inter-laboratory consistency and regulatory compliance. Furthermore, standardized bioinformatics workflows and regularly updated reference databases complement wet-lab validation and collectively underpin robust mNGS diagnostics.

Regarding turnaround time, traditional mNGS can reduce processing to less than 24 h, substantially improving on the 6–8 weeks typical of culture. Yet, it is slower than targeted molecular platforms such as Xpert MTB/RIF, which deliver results in approximately 2 h. Recently, emerging real-time mNGS platforms based on nanopore sequencing have shortened sequencing to 6–8 h, offering rapid pathogen detection options (51). However, nanopore-based metagenomic methods face challenges like lower throughput and raw data accuracy compared to short-read platforms. These limitations are pronounced when detecting intracellular pathogens with thick cell walls, such as MTBC, and in identifying single-nucleotide drug resistance mutations. To date, no reliable clinical application of nanopore-based mNGS for TB diagnosis has been reported, indicating ongoing technical barriers.

Lastly, clinical interpretation of mNGS data presents major challenges. As discussed earlier in this article, mNGS detects microbial nucleic acid fragments but cannot distinguish live from dead organisms. This complication is intensified when multiple microbes are detected, blurring pathogen versus colonizer differentiation. In immunocompetent patients with typical clinical presentations, identifying the causative pathogen is relatively straightforward. However, in immunosuppressed populations, such as HIV-infected individuals or organ transplant recipients, clinical symptoms are frequently masked, and the presence of opportunistic or polymicrobial infections further complicates the interpretation of diagnostic results. Accurate clinical judgment demands close collaboration between laboratory and clinicians and integration of multiple factors: pathogen pathogenicity and colonization tendencies of detected organisms, local epidemiology characteristics, patient immune status, disease progression, and consistency with other diagnostics (Fig. 1). This multidimensional approach illustrates why identical mNGS results may have differing clinical implications in clinical practice.

## RATIONAL APPLICATION OF MNGS IN TB DIAGNOSIS AT THE CURRENT STAGE

TB manifests with diverse clinical presentations across various populations and infection types, and a definitive diagnosis depends on clinical manifestations, imaging findings, and microbiological evidence. Under the current paradigm in which microbiological evidence remains anchored to microbial culture and histopathology as the gold standards, the application of mNGS in TB diagnosis necessitates prudent selection of appropriate clinical indications (Table 1). For non-critically ill patients suspected of pulmonary TB or for follow-up evaluation of previously diagnosed TB cases, traditional diagnostic methods, such as AF stain, serological tests (T-SPOT.TB), mycobacterial culture, and rapid molecular assays like GeneXpert MTB/RIF, should be prioritized. When traditional tests are negative but TB infection cannot be excluded, or when there is suspicion of co-infection with rare or atypical pathogens, mNGS can be considered for pathogen identification. In cases clinically suggestive of extra-pulmonary TB, such as disseminated TB, tissue or lymph node TB, and urinary tract TB, whose sample types are not FDA cleared for GeneXpert testing, mNGS can be employed as an adjunct diagnostic tool alongside comprehensive traditional examinations. For critically ill patients, immunocompromised hosts, and patients with complex infections of unknown etiology, early application of mNGS for pathogen screening is recommended to enhance diagnostic sensitivity and facilitate targeted interventions. It is important to emphasize that due to the high cost, operational complexity, and inability to distinguish between live and dead organisms, mNGS is not suitable for community TB screening and routine monitoring of treatment efficacy or disease recurrence. Its limited sensitivity also prevents it from accurately detecting MTBC drug resistance genes. Future initiatives should concentrate on technological optimization and multidisciplinary collaboration to further augment the clinical utility of mNGS in TB diagnosis.

## PERSPECTIVE

Overall, the application of mNGS in TB diagnosis embodies a technology with tremendous potential but remains constrained by apparent limitations. Its broad-spectrum pathogen detection capability and high sensitivity significantly enhance detection rates, demonstrating unique clinical value particularly in complex cases such as extra-pulmonary TB and in immunocompromised patients. Nevertheless, the technology faces notable barriers, including complex workflows, lack of standardization, suboptimal turnaround time, and limited ability to interpret results, particularly regarding drug resistance gene identification. Future endeavors should prioritize extensive research to optimize clinically applicable technical protocols. There is also an urgent need for prospective studies to identify patient populations that would benefit most from mNGS testing, define cost-effective clinical scenarios, and develop optimal diagnostic algorithms that incorporate this technology. Furthermore, the development of novel diagnostic paradigms, such as integrated microbial-host models, should be actively pursued to leverage the strengths of mNGS with traditional diagnostic methods, with the ultimate goal of building a more efficient, accurate, and scalable strategy for TB diagnosis.

## References

[1] World Health Organization. 2024. Global tuberculosis report 2024. https://www.who.int/teams/global-programme-on-tuberculosis-and-lung-health/tb-reports/global-tuberculosis-report-2024.

[2] Harries AD, Mphasa NB, Mundy C, Banerjee A, Kwanjana JH, Salaniponi FML. 2000. Screening tuberculosis suspects using two sputum smears. Int J Tuberc Lung Dis 4:36–40. 10654642

[3] Xu P, Yang K, Yang L, Wang Z, Jin F, Wang Y, Feng J. 2022. Next-generation metagenome sequencing shows superior diagnostic performance in acid-fast staining sputum smear-negative pulmonary tuberculosis and non-tuberculous mycobacterial pulmonary disease. Front Microbiol 13:898195. doi:10.3389/fmicb.2022.898195 35847073 PMC9283093

[4] Zhang L, Shi X, Zhang Y, Zhang Y, Huo F, Zhou B, Deng G, Liu X. 2017. Analysis of factors influencing diagnostic accuracy of T-SPOT. TB for active tuberculosis in clinical practice. Sci Rep 7:7764. doi:10.1038/s41598-017-07785-6 28798488 PMC5552688

[5] Pai M, Behr M. 2016. Latent Mycobacterium tuberculosis infection and interferon-gamma release assays. Microbiol Spectr 4. doi:10.1128/microbiolspec.TBTB2-0023-2016 27763261

[6] MacLean E, Kohli M, Weber SF, Suresh A, Schumacher SG, Denkinger CM, Pai M. 2020. Advances in molecular diagnosis of tuberculosis. J Clin Microbiol 58:10–1128. doi:10.1128/JCM.01582-19 PMC7512154 32759357

[7] Quainoo S, Coolen JPM, van Hijum SAFT, Huynen MA, Melchers WJG, van Schaik W, Wertheim HFL. 2017. Whole-genome sequencing of bacterial pathogens: the future of nosocomial outbreak analysis. Clin Microbiol Rev 30:1015–1063. doi:10.1128/CMR.00016-17 28855266 PMC5608882

[8] World Health Organization. 2024. WHO consolidated guidelines on tuberculosis: module 3: diagnosis: rapid diagnostics for tuberculosis detection. 3rd ed. https://www.who.int/publications/i/item/9789240089488. 38527162

[9] Han D, Li Z, Li R, Tan P, Zhang R, Li J. 2019. mNGS in clinical microbiology laboratories: on the road to maturity. Crit Rev Microbiol 45:668–685. doi:10.1080/1040841X.2019.1681933 31691607

[10] Filkins LM, Bryson AL, Miller SA, Mitchell SL. 2020. Navigating clinical utilization of direct-from-specimen metagenomic pathogen detection: clinical applications, limitations, and testing recommendations. Clin Chem 66:1381–1395. doi:10.1093/clinchem/hvaa183 33141913

[11] Han D, Li R, Shi J, Tan P, Zhang R, Li J. 2020. Liquid biopsy for infectious diseases: a focus on microbial cell-free DNA sequencing. Theranostics 10:5501–5513. doi:10.7150/thno.45554 32373224 PMC7196304

[12] Carbo EC, Blankenspoor I, Goeman JJ, Kroes ACM, Claas ECJ, De Vries JJC. 2021. Viral metagenomic sequencing in the diagnosis of meningoencephalitis: a review of technical advances and diagnostic yield. Expert Rev Mol Diagn 21:1139–1146. doi:10.1080/14737159.2021.1985467 34607520

[13] Liu D, Zhou H, Xu T, Yang Q, Mo X, Shi D, Ai J, Zhang J, Tao Y, Wen D, et al.. 2021. Multicenter assessment of shotgun metagenomics for pathogen detection. EBioMedicine 74:103649. doi:10.1016/j.ebiom.2021.103649 34814051 PMC8608867

[14] Diao Z, Zhao Z, Han Y, Chen Y, Huang T, Feng L, Ma Y, Li J, Zhang R. 2025. A comprehensive assessment of metagenomic cfDNA sequencing for microbe detection. Clin Chem 71:763–774. doi:10.1093/clinchem/hvaf044 40272442

[15] Han D, Diao Z, Lai H, Han Y, Xie J, Zhang R, Li J. 2022. Multilaboratory assessment of metagenomic next-generation sequencing for unbiased microbe detection. J Adv Res 38:213–222. doi:10.1016/j.jare.2021.09.011 35572414 PMC9091723

[16] You Y, Ni YM, Shi G. 2024. Diagnostic accuracy of metagenomic next-generation sequencing in pulmonary tuberculosis: a systematic review and meta-analysis. Syst Rev 13:317. doi:10.1186/s13643-024-02733-8 39731100 PMC11674177

[17] Liu X, Chen Y, Ouyang H, Liu J, Luo X, Huang Y, Chen Y, Ma J, Xia J, Ding L. 2021. Tuberculosis diagnosis by metagenomic next-generation sequencing on bronchoalveolar lavage fluid: a cross-sectional analysis. Int J Infect Dis 104:50–57. doi:10.1016/j.ijid.2020.12.063 33359946

[18] Xiong W, Dong L, Zhu N, Zhou D, Li S, Lv J, Xu M, Zhang Y, Li S. 2025. The diagnostic value of metagenomic next-generation sequencing in suspected pulmonary tuberculosis patients with scarce sputum or negative sputum etiological test results. Diagn Microbiol Infect Dis 111:116633. doi:10.1016/j.diagmicrobio.2024.116633 39644540

[19] Xiao H, Zhou C, Xiao Z, Cai F, Zhang S, Sheng S, Jin C, Fu Y. 2025. Metagenomic next-generation sequencing of bronchoalveolar lavage fluid samples offers diagnostic utility in bacteriologically negative pulmonary tuberculosis. Diagn Microbiol Infect Dis 111:116725. doi:10.1016/j.diagmicrobio.2025.116725 39954395

[20] Ou Y, Li D, Long X, He H, Qing L, Tian Y, Ren J, Zhou Q, Tan Y. 2025. Study on the early diagnostic value of nanopore sequencing in alveolar lavage fluid smear-negative pulmonary tuberculosis. Braz J Microbiol 56:365–372. doi:10.1007/s42770-024-01575-9 39621292 PMC11885687

[21] Jin W, Pan J, Miao Q, Ma Y, Zhang Y, Huang Y, Yao Y, Su Y, Wang Q, Wang M, Li B, Bao R, Gao X, Wu H, Hu B. 2020. Diagnostic accuracy of metagenomic next-generation sequencing for active tuberculosis in clinical practice at a tertiary general hospital. Ann Transl Med 8:1065. doi:10.21037/atm-20-2274 33145284 PMC7575944

[22] Fu M, Cao L-J, Xia H-L, Ji Z-M, Hu N-N, Leng Z-J, Xie W, Fang Y, Zhang J-Q, Xia D-Q. 2022. The performance of detecting Mycobacterium tuberculosis complex in lung biopsy tissue by metagenomic next-generation sequencing. BMC Pulm Med 22:288. doi:10.1186/s12890-022-02079-8 35902819 PMC9330940

[23] Wang S, Chen Y, Wang D, Wu Y, Zhao D, Zhang J, Xie H, Gong Y, Sun R, Nie X, Jiang H, Zhang J, Li W, Liu G, Li X, Huang K, Huang Y, Li Y, Guan H, Pan S, Hu Y. 2019. The feasibility of metagenomic next-generation sequencing to identify pathogens causing tuberculous meningitis in cerebrospinal fluid. Front Microbiol 10:1993. doi:10.3389/fmicb.2019.01993 31551954 PMC6733977

[24] Sun W, Lu Z, Yan L. 2021. Clinical efficacy of metagenomic next-generation sequencing for rapid detection of Mycobacterium tuberculosis in smear-negative extrapulmonary specimens in a high tuberculosis burden area. Int J Infect Dis 103:91–96. doi:10.1016/j.ijid.2020.11.165 33227518

[25] Ma J, Jiang Y, He Y, Zhou H. 2024. The value of metagenomic next-generation sequencing with blood samples for the diagnosis of disseminated tuberculosis. Front Cell Infect Microbiol 14:1456119. doi:10.3389/fcimb.2024.1456119 39717545 PMC11663735

[26] Yuan J, Wang L, Zhang W, Deng C, Li Q, Meng Y, Chen Y. 2024. Performance of metagenomic Next-Generation Sequencing and metagenomic Nanopore Sequencing for the diagnosis of tuberculosis in HIV-positive patients. Front Cell Infect Microbiol 14:1423541. doi:10.3389/fcimb.2024.1423541 39233907 PMC11371759

[27] Zhou H, Pei Y, Xie Q, Nie W, Liu X, Xia H, Jiang J. 2024. Diagnosis and insight into the unique lung microbiota of pediatric pulmonary tuberculosis patients by bronchoalveolar lavage using metagenomic next-generation sequencing. Front Cell Infect Microbiol 14:1492881. doi:10.3389/fcimb.2024.1492881 39748884 PMC11693512

[28] Yuan J, Ma L, Du J, Sun H, Li S, Zhou G, Rao G, Sun F, Chen W, Miao H, Tian D, Cheng C, Wang Y, Li L, Li L, Pang Y. 2025. Host DNA depletion assisted metagenomic sequencing of bronchoalveolar lavage fluids for diagnosis of pulmonary tuberculosis. Ann Clin Microbiol Antimicrob 24:13. doi:10.1186/s12941-025-00782-y 39962548 PMC11834276

[29] Miao Q, Ma Y, Wang Q, Pan J, Zhang Y, Jin W, Yao Y, Su Y, Huang Y, Wang M, Li B, Li H, Zhou C, Li C, Ye M, Xu X, Li Y, Hu B. 2018. Microbiological diagnostic performance of metagenomic next-generation sequencing when applied to clinical practice. Clin Infect Dis 67:S231–S240. doi:10.1093/cid/ciy693 30423048

[30] Blauwkamp TA, Thair S, Rosen MJ, Blair L, Lindner MS, Vilfan ID, Kawli T, Christians FC, Venkatasubrahmanyam S, Wall GD, Cheung A, Rogers ZN, Meshulam-Simon G, Huijse L, Balakrishnan S, Quinn JV, Hollemon D, Hong DK, Vaughn ML, Kertesz M, Bercovici S, Wilber JC, Yang S. 2019. Analytical and clinical validation of a microbial cell-free DNA sequencing test for infectious disease. Nat Microbiol 4:663–674. doi:10.1038/s41564-018-0349-6 30742071

[31] Bosquez JM, Graf EH. 2025. Reducing the noise in plasma metagenomics to further define clinical utility. Clin Chem 71:728–730. doi:10.1093/clinchem/hvaf065 40415580

[32] Singh H, Gonzalez-Juarbe N, Pieper R, Yu Y, Vashee S. 2024. Predictive biomarkers for latent Mycobacterium tuberculosis infection. Tuberculosis (Edinb) 147:102399. doi:10.1016/j.tube.2023.102399 37648595 PMC10891298

[33] Shi R, Wang Y, Zhou S, Zhang Y, Zheng S, Zhang D, Du X, Gu W, Xu Y, Zhu C. 2023. Metagenomic next-generation sequencing for detecting lower respiratory tract infections in sputum and bronchoalveolar lavage fluid samples from children. Front Cell Infect Microbiol 13:1228631. doi:10.3389/fcimb.2023.1228631 37662001 PMC10470636

[34] Ai J-W, Li Y, Cheng Q, Cui P, Wu H-L, Xu B, Zhang W-H. 2018. Diagnosis of local hepatic tuberculosis through next-generation sequencing: smarter, faster and better. Clin Res Hepatol Gastroenterol 42:178–181. doi:10.1016/j.clinre.2018.04.007 29759945

[35] Benoit P, Brazer N, de Lorenzi-Tognon M, Kelly E, Servellita V, Oseguera M, Nguyen J, Tang J, Omura C, Streithorst J, Hillberg M, Ingebrigtsen D, Zorn K, Wilson MR, Blicharz T, Wong AP, O’Donovan B, Murray B, Miller S, Chiu CY. 2024. Seven-year performance of a clinical metagenomic next-generation sequencing test for diagnosis of central nervous system infections. Nat Med 30:3522–3533. doi:10.1038/s41591-024-03275-1 39533109 PMC11645279

[36] Tang H, Chen Y, Tang X, Wei M, Hu J, Zhang X, Xiang D, Yang Q, Han D. 2025. Yield of clinical metagenomics: insights from real-world practice for tissue infections. EBioMedicine 111:105536. doi:10.1016/j.ebiom.2024.105536 39729886 PMC11732148

[37] Wang G, Wang S, Jiang G, Yang X, Huang M, Huo F, Ma Y, Dai G, Li W, Chen X, Huang H. 2019. Xpert MTB/RIF Ultra improved the diagnosis of paucibacillary tuberculosis: a prospective cohort study. Journal of Infection 78:311–316. doi:10.1016/j.jinf.2019.02.010 30796951

[38] Balcha TT, Sturegård E, Winqvist N, Skogmar S, Reepalu A, Jemal ZH, Tibesso G, Schön T, Björkman P. 2014. Intensified tuberculosis case-finding in HIV-positive adults managed at Ethiopian health centers: diagnostic yield of Xpert MTB/RIF compared with smear microscopy and liquid culture. PLoS One 9:e85478. doi:10.1371/journal.pone.0085478 24465572 PMC3899028

[39] Guo D, Fan J, Zhang X, Chen S, Du X. 2025. Next-generation sequencing assistance in the diagnosis of active tuberculosis following allogeneic hematopoietic stem cell transplantation: a case series. J Infect Chemother 31:102683. doi:10.1016/j.jiac.2025.102683 40118380

[40] Thomas TA. 2017. Tuberculosis in children. Pediatr Clin North Am 64:893–909. doi:10.1016/j.pcl.2017.03.010 28734517 PMC5555046

[41] Reuter A, Hughes J, Furin J. 2019. Challenges and controversies in childhood tuberculosis. Lancet 394:967–978. doi:10.1016/S0140-6736(19)32045-8 31526740

[42] World Health Organization. 2024. WHO bacterial priority pathogens list, 2024: bacterial pathogens of public health importance to guide research, development and strategies to prevent and control antimicrobial resistance. Available from: https://www.who.int/publications/i/item/9789240093461. Retrieved 22 Aug 2025.

[43] World Health Organization. 2023. The use of next-generation sequencing for the surveillance of drug-resistant tuberculosis: an implementation manual. Available from: https://www.who.int/publications/i/item/9789240078079. Retrieved 22 Aug 2025.

[44] Cohen KA, Manson AL, Desjardins CA, Abeel T, Earl AM. 2019. Deciphering drug resistance in Mycobacterium tuberculosis using whole-genome sequencing: progress, promise, and challenges. Genome Med 11:45. doi:10.1186/s13073-019-0660-8 31345251 PMC6657377

[45] Schwab TC, Perrig L, Göller PC, Guebely De la Hoz FF, Lahousse AP, Minder B, Günther G, Efthimiou O, Omar SV, Egger M, Fenner L. 2024. Targeted next-generation sequencing to diagnose drug-resistant tuberculosis: a systematic review and meta-analysis. Lancet Infect Dis 24:1162–1176. doi:10.1016/S1473-3099(24)00263-9 38795712 PMC11881551

[46] Kalantar KL, Neyton L, Abdelghany M, Mick E, Jauregui A, Caldera S, Serpa PH, Ghale R, Albright J, Sarma A, Tsitsiklis A, Leligdowicz A, Christenson SA, Liu K, Kangelaris KN, Hendrickson C, Sinha P, Gomez A, Neff N, Pisco A, Doernberg SB, Derisi JL, Matthay MA, Calfee CS, Langelier CR. 2022. Integrated host-microbe plasma metagenomics for sepsis diagnosis in a prospective cohort of critically ill adults. Nat Microbiol 7:1805–1816. doi:10.1038/s41564-022-01237-2 36266337 PMC9613463

[47] Mick E, Tsitsiklis A, Kamm J, Kalantar KL, Caldera S, Lyden A, Tan M, Detweiler AM, Neff N, Osborne CM, Williamson KM, Soesanto V, Leroue M, Maddux AB, Simões EA, Carpenter TC, Wagner BD, DeRisi JL, Ambroggio L, Mourani PM, Langelier CR. 2023. Integrated host/microbe metagenomics enables accurate lower respiratory tract infection diagnosis in critically ill children. J Clin Invest 133:e165904. doi:10.1172/JCI165904 37009900 PMC10065066

[48] Ramachandran PS, Ramesh A, Creswell FV, Wapniarski A, Narendra R, Quinn CM, Tran EB, Rutakingirwa MK, Bangdiwala AS, Kagimu E, et al.. 2022. Integrating central nervous system metagenomics and host response for diagnosis of tuberculosis meningitis and its mimics. Nat Commun 13:1675. doi:10.1038/s41467-022-29353-x 35354815 PMC8967864

[49] Xu F, Wang Q, Zhang N, Xing X, Liu Z, Li K, Ma Y, Ou Q, Jia Y, Chen X, Zhang C, Pan J, Che N. 2023. Simultaneous diagnosis of tuberculous pleurisy and malignant pleural effusion using metagenomic next-generation sequencing (mNGS). J Transl Med 21:680. doi:10.1186/s12967-023-04492-x 37777783 PMC10541691

[50] Han D, Yu F, Lou B, Yang B, Shen Y, Liu H, Tang H, Zhou H, Zheng S, Chen Y. 2025. Multimodal metagenomic profiling of bronchoalveolar lavage fluid for diagnostic classification of pulmonary diseases. Res Square. doi:10.21203/rs.3.rs-6108429/v1

[51] Gu W, Deng X, Lee M, Sucu YD, Arevalo S, Stryke D, Federman S, Gopez A, Reyes K, Zorn K, Sample H, Yu G, Ishpuniani G, Briggs B, Chow ED, Berger A, Wilson MR, Wang C, Hsu E, Miller S, DeRisi JL, Chiu CY. 2021. Rapid pathogen detection by metagenomic next-generation sequencing of infected body fluids. Nat Med 27:115–124. doi:10.1038/s41591-020-1105-z 33169017 PMC9020267

